# Periprosthetic joint infection of megaprostheses for oncologic and non-oncologic indications—IMPLANT retention or removal? A retrospective cohort study of 50 cases

**DOI:** 10.1186/s42836-025-00314-1

**Published:** 2025-06-05

**Authors:** Benjamin Schlossmacher, Elena Strasser, Vincent Lallinger, Florian Pohlig, Ruediger von Eisenhart-Rothe, Igor Lazic

**Affiliations:** Department of Orthopaedics and Sports Orthopaedics, Klinikum rechts der Isar, School of Medicine, TUM Universitaetsklinikum, Ismaninger Str. 22, München, 81675 Germany

**Keywords:** Arthroplasty, Periprosthetic joint infection, Megaprostheses, Revision arthroplasty, DAIR

## Abstract

**Background and purpose:**

Periprosthetic joint infection (PJI) is a devastating but rare complication. Its incidence ranges between 1%–2% in primary arthroplasties. However, infection rates are much higher in megaprostheses (15%–43%). Revision of megaprostheses (MP) is a highly complex procedure associated with massive bone loss, so that implant retention occurs as a viable initial therapy option even in chronic infections. Unfortunately, literature regarding therapy strategies and outcome reports for PJI in MP is scarce. Reinfection rates are reported to be between 22 and 58%. We therefore proposed the following questions: What is the overall outcome of PJI in MP in our cohort, and are there significant differences in infection-free survival between various surgical strategies?

**Methods:**

In this retrospective cohort study, 50 cases of PJI in MP treated from 2010 to 2022 were identified. The median (IQR) age was 70.5 (16.3) years. Mean follow-up was 19.0 months. Treatment outcome was categorized following international consensus criteria.

**Results:**

Overall infection-free implant survival was 42.0%. 7 patients died in direct association with the ongoing PJI, and 7 had to undergo amputation. Two-stage revision had the highest success rate of 71.4% (5/7), followed by multi-stage surgery (57.1%; 4/7), DAIR (38.7%; 12/31), and single-stage revision (0%; 0/5) (*P* = 0.009). Overall, treatment success rates following DAIR were 55.6% (10/18) for acute and 15.4% (2/13) for chronic infections (*P* = 0.027).

The most common pathogens were coagulase-negative Staphylococci (42.0%; 21/50) and Staphylococcus aureus (34.0%; 17/50). Gram-negative pathogens accounted for 16.0% (8/50).

**Conclusions:**

PJI in MP remains a devastating complication with low success rates. Two-stage revision is the most promising treatment option, but it requires patients to be able to cope with the burden of multiple surgeries. DAIR cannot be recommended as a definitive treatment for chronic cases (15% success rate) and should be questioned in acute cases (56% success rate), as infection eradication is rare. DAIR can be considered a low-impact surgery for infection control if more extensive surgery is not viable.

Video Abstract

**Supplementary Information:**

The online version contains supplementary material available at 10.1186/s42836-025-00314-1.

## Introduction

Due to the continual rise in total joint arthroplasty (TJA) rates in recent years, despite generally favorable outcomes, there is a notable incidence of complications, leading to revision rates of up to 20% [1, 2].

In a few distinct cases where large osseous defects occur due to either extensive tumor resection or multiple revision arthroplasties, the only viable option for limb salvage is the use of a megaprostheses (MP).

The implantation of an MP itself is a highly complex procedure that is often associated with major restrictions for the patient and high complication rates. Nevertheless, it has become a viable option with promising results in recent years. Still, the most devastating complication is a periprosthetic joint infection.

One-, two-, or multi-stage concepts with implant removal were developed for proper infection eradication in chronic PJI. Debridement, antibiotics, and implant retention (DAIR) is usually reserved for cases of acute infection, as the theory proposes that an intact biofilm has not yet formed and can still be removed by appropriate debridement and antibiotic therapy [3].

Few data are available concerning therapy options for PJI in MP, and evidence-based therapeutic algorithms are lacking [4–6].

Existing treatment algorithms usually recommend the exchange of the implant in cases of chronic PJI, with multiple options regarding the exact number of stages [7].

Although DAIR seems to be associated with higher rates of persisting or recurrent PJI in MP, implant retention is a fallback option if perioperative risks are too high or another implant exchange is not feasible due to limited bone stock and low chances of reimplantation [8, 9].

In this study, we analysed the outcome of PJI in MP at our institution regarding surgical treatment options and the type of infection (acute vs. chronic) to further compare the roles of implant retention and removal.

## Methods

### Study design

This retrospective cohort study included 53 consecutive cases of PJI in MP treated at an academic tertiary referral center between 2010 and 2022. All data were drawn retrospectively from a comprehensive database for all PJI treated at our institution. All eligible cases were included. Patients were followed up either through regular outpatient clinic visits or phone calls if a visit was not feasible. 3 cases had no follow-up after treatment and were therefore excluded. 50 cases met final inclusion criteria.

The study was approved by the local institution’s Ethics Committee (reference no. 714/20 S) and was conducted in accordance with the Helsinki Declaration.

### Patients/implant selection

The primary indication for MP was major bone loss following extensive tumor resection, aseptic loosening, periprosthetic fracture, or previous PJI. Implants that were defined as MP were proximal and distal femoral replacements (PFR/DFR) and (intramedullary) total femoral replacements (IFR/TFR), as well as large cranial socket cups with intra- and extramedullary iliac fixation and custom-made acetabular implants for distinct osseous defects.

### Treatment algorithm

Prior to revision of the implant, a detailed case assessment took place, and treatment options were discussed with an interdisciplinary team as well as the patient. The team members were the responsible orthopedic surgeon, a microbiologist, and a pharmacist for interdisciplinary decision making.

Cases were divided into acute and chronic infections (more or less than four weeks of symptoms) and following the classification of Tsukayama et al. [10].

Surgical therapy included debridement, antibiotics, and implant retention (DAIR) or implant removal through a single-, two-, or multi-stage procedure. All staged procedures involved the use of an antibiotic-loaded spacer. Multi-stage was defined as at least one repeated spacer-exchange between the removal of the MP and reimplantation.

In accordance with our institution’s PJI algorithm, indications for DAIR were short symptom durations, ideally less than two weeks, but often extended up to four weeks. For implant removal, assumed chronic infections or septic loosening are the most frequently used indications. For the following cohort of MP infections, treatment decisions were mostly based on individual discussion and therefore did not follow the regular algorithm at all costs, especially when attempting to retain the implant.

### Variables and outcome measures

General patients’ demographics were collected and analyzed for significant differences between groups of interest.

Cases were classified according to the JS-BACH classification by Hotchen et al. [11]. Due to the presence of an MP, all cases were classified as limited options.

Patients’ health status was summarized in the form of the Charlson comorbidity score (CCS).

Treatment outcome was categorized following the 2013 Delphi consensus as well as the 2019 Fillingham classification (MSIS) [12, 13]. The primary endpoint was infection-free implant survival, which was defined as a healed wound without fistula, drainage, or pain, and no infection recurrence with or without septic revision surgery. Success with suppressive antimicrobial therapy and death due to PJI were recorded as secondary endpoints.

### Statistics

Normally distributed variables are presented as mean and standard deviation (SD), non-normally distributed variables as median and interquartile range (IQR). The Shapiro–Wilk Test was used to assess whether the variables followed a normal distribution.

For non-normally distributed variables, the Mann–Whitney-U test and t-test for normally distributed variables were performed for all continuous variables. Pearson’s Chi-Squared test was performed for comparison of categorial variables. Values of α < 0.05 were considered to indicate statistical significance. Survival analysis was performed via Kaplan–Meier survival statistics. Log-rank test and cox-regression were used for survival comparisons. Statistical analysis was carried out using IBM SPSS Statistics for Windows, version 27.0 (IBM Corporation, Armonk, NY, USA).

## Results

The median (IQR) age at surgery was 70.5 (16.3) years. 42.0% of patients were female. 24 acute and 26 chronic infections were included. The mean (SD) follow-up was 19.0 (24.6) months. There were no significant differences regarding epidemiologic factors between the acute and chronic groups.

Detailed patient demographics, data on implants, and indications are shown in Table 1.

**Table 1:** Patient demographics

	**All cases** **(*n* = 50)**	**Acute infection**^**a**^ **(*n* = 24)**	**Chronic infection**^**b**^ **(*n* = 26)**	***P*-Value**
Age in years (median; IQR)	70.5 (16.3)	73.0 (23.3)	70.0 (18.3)	0.837
Sex (*n*; %)				0.598
Male	29 (58.0)	13 (54.2)	16 (61.5)	
Female	21 (42.0)	11 (45.8)	10 (38.5)	
Follow-Up in months (mean; SD)	19.0 (24.6)	16.8 (17.8)	21.0 (29.7)	0.558
ASA classification (*n*; %)				0.476
ASA I	1 (2.0)	1 (4.2)	0	
ASA II	20 (40.0)	10 (41.7)	10 (38.5)	
ASA III	28 (56.0)	12 (50.0)	16 (61.5)	
ASA IV	1 (2.0)	1 (4.2)	0	
BMI in kg/m^2^ (median; IQR)	28.7 (7.5)	29.2 (10.7)	28.0 (7.0)	0.340
Comorbidities (*n*; %)				
CCS^c^	4.9 (2.6)	5.3 (2.2)	4.5 (2.9)	0.270
Anemia (Hb < 12 g/dL)	37 (74.0)	18 (75.0)	19 (73.1)	
Diabetes mellitus	14 (29.2)	7 (29.2)	7 (26.9)	
Cancer	23 (47.9)	13 (54.2)	10 (41.7)	
COPD	7 (14.6)	3 (12.5)	4 (15.4)	
Chronic renal failure	11 (22.9)	7 (29.2)	4 (15.4)	
Rheumatoid arthritis	2 (4.2)	1 (4.2)	1 (3.8)	
Liver damage	5 (10.4)	2 (8.3)	3 (12.5)	
Obesity (BMI > 30 kg/m^2^)	19 (38.0)	11 (45.8)	8 (30.8)	
Corticoids	3 (6.3)	1 (4.2)	2 (7.7)	
Implants (*n*; %)				0.548
Proximal femoral replacement	14 (28.0)	9 (37.5)	5 (19.2)	
Distal femoral replacement	13 (26.0)	6 (25.0)	7 (26.9)	
Intramedullary femoral replacement	4 (8.0)	2 (8.3)	2 (7.7)	
Total femoral replacement	8 (16.0)	2 (8.3)	6 (23.1)	
Cranial socket cup with flange and peg	5 (10.0)	3 (12.5)	2 (7.7)	
Custom-made acetabular revision implant	6 (12.0)	2 (8.3)	4 (15.4)	
Prior surgeries (median; IQR)	4.0 (4.0)	3.5 (2.8)	4.5 (3.3)	0.262
Indication for MP^d^ (*n*; %)				0.825
PJI^e^	19 (38.0)	9 (37.5)	10 (38.5)	
Aseptic loosening	13 (26.0)	5 (20.8)	8 (30.8)	
Oncologic	11 (22.0)	6 (25.0)	5 (19.2)	
Fracture	7 (14.0)	4 (16.7)	3 (11.5)	

^a^Acute infection: < 4 weeks of symptom duration

^b^Chronic infection: > 4 weeks of symptom duration

^c^*CCS* Charlson Comorbidity Score

^d^*MP* megaprosthesis

^e^*PJI* periprosthetic joint infection

Regarding the microbial spectrum, coagulase-negative staphylococci were the most common pathogen (42.0%; 21/50), followed by Staphylococcus aureus (34.0%; 17/50). Gram-negative bacteria (16.0%; 8/50) and streptococci (12.0%; 6/50) were common, as well. Polymicrobial infections were found in 11 cases (28.0%). The mean (SD) duration of antimicrobial therapy was 11.3 weeks (5.7). 10 patients presented with a preoperative fistula.

Further details on the microbial spectrum and antibiotic therapy can be found in Table 4 in Appendix.

DAIR was the most frequently performed surgery in 31 cases (62.0%), followed by two- and multi-stage revision with 7 cases (18.0%), and one-stage revision with 5 cases (12.8%), respectively. In 15 cases, a lavage of the affected joint or implant was performed before definitive treatment (30.0%) to reduce the load of pathogens in acute septic cases.

### Overall outcome and risk factors

Overall infection-free survival was 42.0% (21/50) after a mean of 19.0 months.

In 7 cases, an amputation of the affected extremity had to be performed as definitive treatment for uncontrollable infection (14.0%). 13 patients died during the observed period. In 7 cases, the cause of death was directly associated with the ongoing PJI (14.0%).

Gram-negative bacteria (GN) had a significantly worse success rate than coagulase-negative staphylococci (CNS) (0/4 vs. 8/21; *P* = 0.036). GN showed the worst overall outcome, with a 100% failure rate, irrespective of the surgical approach.

The type of implant, polymicrobial infections, type of antibiotics, type of infection (acute vs. chronic; Tsukayama), presence of a preoperative fistula, and CCS were no significant predictors for treatment success following regression analysis.

The indication for initial MP was no significant predictor, either. When a PJI was the indication for implantation of the first MP, the final success rate of infection-free survival was 42.1% compared to 48.4% for aseptic indications (*P* = 0.665).

Considering oncologic and non-oncologic primary MP indications, success rates were 41.7% and 47.4%, respectively (*P* = 0.730).

### Comparison of treatment options

Two-stage revision had the highest success rate of 71.4% (5/7), followed by multi-stage surgery (57.1%; 4/7). DAIR was associated with an infection-free survival rate of 38.7% (12/31). Single-stage revision was the worst, with a 0% success rate (0/5).

**Fig. 1:**
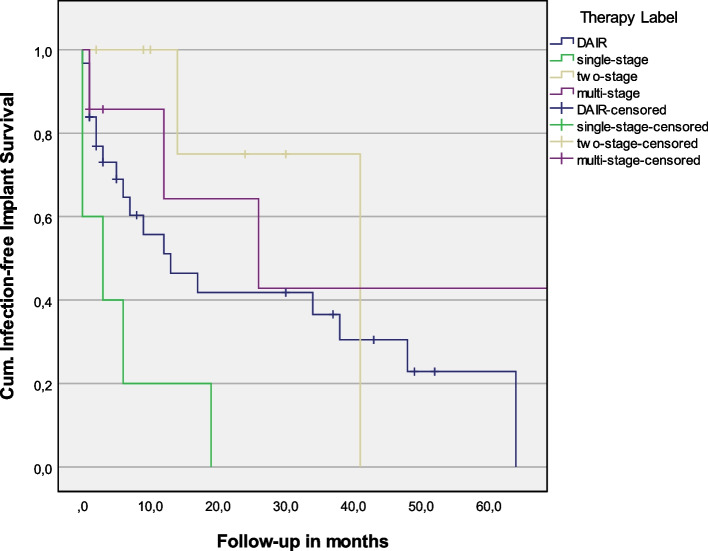
Comparison of Kaplan–Meier infection-free survival between all treatment options (*P* = 0.009). Hazard ratio with DAIR as base-line was 3.0 (95%-CI 1.1–8.5) for one-stage, 0.4 (95%-CI 0.1–1.7) for two-stage, and 0.5 (95%-CI 0.1–1.7) for multi-stage revision

The mean infection-free survival was 26.4 months (95%-CI 15.9–36.9) for DAIR, 6.4 months (95%-CI 0–12.9) for single-stage, 34.2 months (95%-CI 18.0–50.5) for two-stage, and 56.2 months (95%-CI 17.4–94.9) for multi-stage revision (*P* = 0.009; Fig. 1).

Detailed treatment and outcome measures are shown in Tables 2 and 3.

**Table 2:** Treatment and clinical outcome according to the type of infection

	**All cases** **(*n* = 50)**	**Acute infection** **(*n* = 24)**	**Chronic infection** **(*n* = 26)**	***P*-value**
DELPHI-consensus criteria [9]				
No fistula, drainage, pain, or recurrence of infection	21 (42.0)	12 (50.0)	9 (34.6)	0.271
No subsequent surgery for infection	32 (64.0)	17 (70.8)	15 (57.7)	0.258
No PJI-related mortality	43 (86.0)	20 (83.3)	23 (88.5)	0.602
Mean infection-free survival (months; mean; 95%-CI)	35.2 (21.5–48.9)	27.1 (17.6–36.6)	31.9 (15.4–48.4)	0.597
Mean infection-free survival after DAIR (months; mean; 95%-CI)	-	30.7 (19.8–41.6)	15.4 (2.3–28.5)	0.068
Fillingham-classification [11]				
1 - Success, no antibiotics	18 (36.0)	12 (50.0)	6 (23.1)	
2 - Success, antibiotic suppression	3 (6.0)	0	3 (11.5)	
3 A - Aseptic revision > 1y	2 (4.0)	0	2 (7.7)	
3B - Septic revision > 1y	9 (18.0)	3 (12.5)	6 (23.1)	
3 C - Aseptic revision < 1y	1 (2.0)	0	1 (3.8)	
3D - Septic revision < 1y	7 (14.0)	4 (16.7)	3 (11.5)	
3E - Amputation, arthrodesis, resection	7 (14.0)	4 (16.7)	3 (11.5)	
4 A - Death < 1y after PJI	11 (22.0)	5 (20.8)	6 (23.1)	
4B - Death > 1y after PJI	2 (4.0)	1 (4.2)	1 (3.8)	
5 - Fistula	9 (18.0)	2 (8.3)	7 (26.9)	
Death				
Any cause	13 (26.0)	5 (20.8)	8 (30.8)	0.424
PJI-related	7 (14.0)	4 (16.7)	3 (11.5)	0.602

*DAIR* Debridement, antibiotics, and implant retention

**Table 3:** Outcome according to the type of therapy

	**Overall success-rate** **(*n* = 50)**	**DAIR success-rate** **(*n* = 31)**	**1-stage success-rate** **(*n* = 5)**	**2-stage success-rate** **(*n* = 7)**	**Multi-stage success-rate** **(*n* = 7)**	***P*-value**
Overall	21/50 (42.0)	12/31 (38.7)	0/5 (0.0)	5/7 (71.4)	4/7 (57.1)	0.075
Implant type						
Proximal femoral replacement	7/14 (50.0)	6/11 (54.5)	0/1 (0.0)	1/2 (50.0)	-	0.580
Distal femoral replacement	4/13 (30.8)	0/3 (0.0)	0/3 (0.0)	3/4 (75.0)	1/3 (33.3)	0.096
Intramedullary femoral replacement	3/4 (75.0)	1/2 (50.0)	-	1/1 (100.0)	1/1 (100.0)	0.513
Total femoral replacement	3/8 (37.5)	2/6 (33.3)	0/1 (0.0)	-	1/1 (100.0)	0.315
Cranial socket cup with flange and peg	2/5 (40.0)	2/5 (40.0)	-	-	-	-
Custom-made acetabular revision implant	2/6 (33.3)	1/4 (25.0)	-	-	1/2 (50.0)	0.540
Indication						
PJI	7/19 (36.8)	3/10 (30.0)	0/3 (0.0)	2/3 (66.7)	2/3 (66.7)	0.236
Aseptic loosening	7/13 (53.8)	5/10 (50.0)	-	2/2 (100.0)	0/1 (0.0)	0.230
Fracture	3/7 (42.9)	2/4 (50.0)	0/1 (0.0)	0/1 (0.0)	1/1 (100.0)	0.405
Tumour	4/11 (36.4)	2/7 (28.6)	0/1 (0.0)	1/1 (100.0)	1/2 (50.0)	0.446
Non-oncologic (combined)	16/38 (42.1)	10/24 (41.7)	0/4 (0.0)	4/6 (66.7)	2/4 (50.0)	0.212
Type of infection						
Early acute	7/11 (63.6)	6/10 (60.0)	-	1/1 (100.0)	-	0.428
Late acute	5/13 (38.5)	4/8 (50.0)	0/3 (0.0)	1/1 (100.0)	0/1 (0.0)	0.208
Chronic	9/26 (34.6)	2/13 (15.4)	0/2 (0.0)	3/5 (60.0)	4/6 (66.7)	0.080

Significant differences were present between single-stage revision and DAIR (*P* = 0.037), single- and two-stage revision (*P* = 0.005), and single- and multi-stage revision (*P* = 0.004).

### Implant retention in acute and chronic infections

**Fig. 2:**
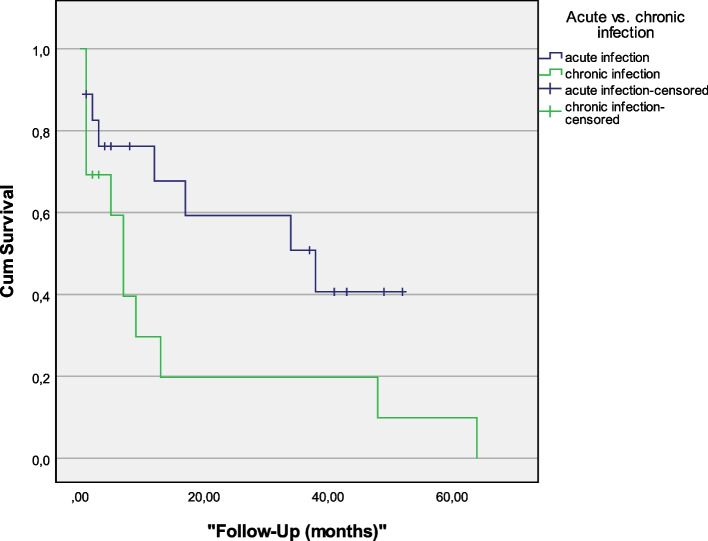
Comparison of Kaplan–Meier infection-free survival between acute and chronic infections (more or less than 4 symptomatic weeks) following DAIR (*P* = 0.027). Hazard ratio for chronic PJI was 2.2 (95%-CI 0.9–5.7)

In terms of infection-free survival after DAIR for acute and chronic infections, a significant difference was found. Overall, treatment success rates following DAIR were 55.6% (10/18) for acute PJI and 15.4% (2/13) for chronic PJI. Mean infection-free survival following Kaplan–Meier analysis was 30.7 months (95%-CI 19.8–41.6) for acute and 15.4 months (95%-CI 2.3–28.5) for chronic infections (*P* = 0.068; Fig. 2).

## Discussion

The diagnosis of PJI in megaprostheses remains a devastating complication after revision-TJA or extensive tumor resection. The first finding of this study is that the infection eradication of PJI in MP is poor regardless of the surgical procedure. In our study, the overall infection-free survival rate was 42.0%. A total of 14.0% of patients had to undergo amputation as salvage treatment for uncontrollable infection, and PJI-related death occurred in 14.0% of cases. These results emphasize the drastic consequences for patients who suffer from an infection of their MP.

The literature on infection eradication in MP is scarce, especially in non-oncologic indications. Previous results demonstrated poor overall outcomes with reinfection rates ranging between 22 and 58% and lower success rates for oncologic indications [14–19]. Possible explanations for the higher PJI relapse rate in tumor patients, despite the younger age, are adjuvant oncologic treatments such as local radiotherapy or systemic chemotherapy, with a consequently compromised immune system. Despite these prior findings, we did not find significant differences between oncologic and non-oncologic indications in our cohort. Oncologic patients underwent regular adjuvant treatment regimens with either local radiotherapy, systemic chemotherapy, or both combined. The previously assumed risk factors for higher failure rates were therefore present and may have caused the slightly lower, but non-significant, success rate in oncologic indications. Except for a significantly lower age, no differences were demographic differences were present between the oncologic and non-oncologic groups.

### Comparison between treatment options

The wide range of success rates can be attributed to various factors: different indications for MP, different definitions of success, and, above all, different surgical approaches. A particular emphasis of this study was on the therapy outcomes depending on the surgical approach.

Two- and multi-stage revision yielded the best results for chronic and acute infections in our cohort. With this approach, two-thirds of either chronic or acute infections were successfully treated, which is in line with previously reported results on PJI MP outcomes [19, 20].

Sigmund et al. demonstrated that two-stage revision with complete removal of the MP showed the best results for the treatment of PJI in MP in musculoskeletal tumor surgery compared to DAIR or single-stage revision.

The authors reported a reinfection probability of approximately 30% at 2 years for PJI in MP [19].

Similarly, Smith et al. investigated the survivorship of MP in revision hip and knee arthroplasty for non-oncologic indications and reported approximately 31% septic complications after two-stage revision of PJI in MP [20].

In contrast, single-stage revision was associated with a significantly worse outcome in our study. Similarly, Jeys et al., Funovics et al., and Sigmund et al. all found higher failure rates for single-stage procedures, ranging from 37%–58%. In this context, it is crucial to mention that the gold standard at our institution is a two-stage revision. Single-stage revisions were reserved for patients with poor general condition to avoid prolonged immobilization and the comorbidities associated with a second operation. Patients in this group were the oldest, most obese, had undergone the most previous surgeries, and presented with the highest CCS.

The role of single-stage revision in PJI of MP cannot be conclusively assessed on the basis of these data.

### Does DAIR play a viable role in the treatment of PJI in megaprostheses?

The most important finding of this study is that DAIR is associated with poor infection eradication in acute and especially devastating in chronic infections.

While implant retention is an appealing option in MP, the approximately 55% success rate of DAIR in acute PJI is unsatisfactory and should be carefully considered in shared decision-making with patients, as nearly half of the infections recur within a short period.

However, the results of DAIR in chronic PJI MP are even more daunting. Infections persisted or recurred with near certainty within this cohort. Even PJI caused by coagulase-negative staphylococci showed exceptionally poor success rates in this study, although success rates ranging from 73 to 83% have been reported in the revision of regular-sized implants [3].

Sukhonthamarn et al. reported on the outcome of PJI in MP after DAIR in 27 patients and showed success rates of 65% after 2 years of follow-up [6]. In comparison to our study, treatment failure had been defined as unplanned revision or salvage surgery. Furthermore, there was a relevant difference in the Charlson comorbidity score, whereby significantly fewer healthy patients were treated in the present study (4.9 vs. 1.1).

If being free of unplanned re-revision for PJI is defined as the primary endpoint in this study, success rates between Sukhonthamarn’s and our cohort are almost equal (65% vs. 64%).

Similarly, Asokan et al. reported a success rate of 65% in 14 patients who underwent DAIR for PJI of femoral MP. Although our results are thus consistent with the literature, it can be further specified that especially in morbidly ill, elderly patients, DAIR is associated with a significantly worse outcome. Particularly in chronic PJI, DAIR was more likely to result in higher mortality, more septic revisions, and more fistulas.

If the therapeutic aim is to eradicate the infection, the better results achieved with the two-stage or multi-stage approach should provide an incentive to consider a more aggressive approach in PJI of MP. Unfortunately, in several cases, a two- or multi-stage approach may not be feasible in MP: the remaining bone stock after explantation of the MP does not always allow for secure fixation of further implants, so an implant retaining procedure is inevitably chosen despite the chronic infection.

Eventually, it must be discussed with these patients whether infection eradication is possible at all or whether infection control should be pursued. Given the complex situation of many patients with MP, a paradigm shift is sometimes necessary, and it becomes more obvious why differentiated systems for outcomes exist [12, 13].

Common additions to DAIR procedures are sinus tracts or suppressive antibiotic therapy (SAT), which ultimately may result in adequate quality of life. Heterogeneous success rates following the SAT in PJI have been reported [21]. However, a recent multicenter cohort study demonstrated an infection control with SAT in 50% of patients at 5-year follow-up [22]. Especially in older patients with PJI in MP, infection control over such a period using SAT appears favorable, considering the results shown here. Recently, Klim et al. reported that a stable sinus tract is a functionally acceptable treatment option with a reasonable quality of life in patients with PJI [23].

These additions to DAIR may be preferable over complex PJI treatment with multiple surgeries and an ambiguous prognosis regarding the patient’s ability to ambulate. If the use of suppressive antibiotics or a sinus tract is considered a treatment success, rates would increase from 42 to 64% in this study.

It remains unclear which patients are suitable for a two-stage approach with a high chance of success and which additions to DAIR will benefit patients with PJI MP. Further studies should investigate these patients at risk for infection persistence and low reimplantation rates. Furthermore, appropriate studies on DAIR additions in PJI of MP must be conducted to allow for recommendations.

In recent years, promising results have been reported for PJI treatment using specific bacteriophage treatment in previously assumed non-curable cases [24]. If this form of therapy becomes established, it could lead to a significant improvement in outcome. However, further, more extensive studies are required to identify significant and relevant differences.

### Limitations

Several limitations of this study should be acknowledged. First, the study’s retrospective design inherently has limitations, including potential biases and data collection challenges. Second, the small cohort size limits the statistical power and the generalizability of the findings to a broader patient population, especially when comparing different treatment options. However, it is worth noting that this study benefits from a representative pool of patients, with 50 cases included. The rare use of MP causes the cohort sizes to remain small.

Third, the absence of functional outcome scores and the short follow-up duration restrict our ability to conclude long-term clinical outcomes. However, a relevant number of infections recurred within a short interval, and we therefore decided to also include patients with short follow-up.

Fourth, due to the significant impact of an implant removal of an MP as discussed above, a selection bias in favor of DAIR procedures must be assumed.

Finally, our cohort consists of several different implant types and indications for implantation of an MP (oncologic and non-oncologic), accounting for a heterogeneous study population.

Since the main focus was on the infectiological outcome, no functional outcomes were assessed.

## Conclusions

In conclusion, PJI in megaprostheses remains a devastating complication with low success rates. Two- and multi-stage revisions are the most promising treatment options, but require patients to be able to cope with the burden of multiple surgeries and sufficient bone stock to ensure reimplantation success.

DAIR should be critically discussed in acute and especially in chronic infections. In chronic PJI, infection eradication cannot be presumed. However, DAIR can be considered a low-impact procedure for infection control if extensive surgery is not viable. Further studies on reimplantation success after the removal of MP and the benefits of DAIR additions in MP are necessary.

## Data Availability

The dataset supporting the conclusions of this article has not been published anywhere but can be made available on request.

## Appendix

**Table 4:** Data on infections and antibiotic treatment

	**All cases** **(*****n***** = 50)**	**Acute infection** **(*****n***** = 24)**	**Chronic infection** **(*****n***** = 26)**	***P*****-Value**
Causative bacteria				
Staphylococcus aureus	17 (34.0)	10 (41.7)	7 (26.9)	
Coagulase-negative staphylococci	21 (42.0)	9 (37.5)	12 (46.2)	
Streptococci	6 (12.0)	3 (12.5)	3 (11.5)	
Gram-negative strands	8 (16.0)	3 (12.5)	5 (19.2)	
Anaerobic strands	1 (2.0)	1 (4.2)	0	
Enterococci	3 (6.0)	2 (8.3)	1 (3.8)	
Candida spp.	3 (6.0)	2 (8.3)	1 (3.8)	
Negative cultures	5 (10.0)	2 (8.3)	3 (11.5)	
Polymicrobial infection	14 (28.0)	7 (29.2)	7 (26.9)	0.554
IV Antibiotics at 1 st stage/DAIR				
Vancomycin	28 (56.0)	17 (70.8)	11 (42.3)	
Daptomycin	2 (4.0)	0	2 (7.7)	
Flucloxacillin	9 (18.0)	6 (25.0)	3 (11.5)	
Ampicillin + Sulbactam	12 (24.0)	9 (37.5)	3 (11.5)	
Piperacillin + Tazobactam	2 (4.0)	0	2 (7.7)	
Penicillin G	3 (6.0)	1 (4.2)	2 (7.7)	
Meropenem	8 (8.0)	5 (20.8)	3 (11.5)	
Imipinem	3 (6.0)	2 (8.3)	1 (3.8)	
Rifampicin	7 (14.0)	4 (16.7)	3 (11.5)	
Clindamycin	4 (8.0)	1 (4.2)	3 (11.5)	
Cefalosporine	8 (16.0)	3 (12.5)	5 (19.2)	
Oral antibiotics				
Fluoroquinolones	27 (54.0)	15 (62.5)	12 (46.2)	
Rifampicin	22 (44.0)	14 (58.3)	8 (30.8)	
Clindamycin	3 (6.0)	0	3 (11.5)	
Cotrimoxazol	6 (12.0)	2 (8.3)	4 (15.4)	
Linezolid	4 (8.0)	1 (4.2)	3 (11.5)	
Amoxicillin	4 (8.0)	2 (8.3)	2 (7.7)	T
Doxycyclin	3 (6.0)	2 (8.3)	1 (3.8)	
Fluconazol	3 (6.0)	2 (8.3)	1 (3.8)	
Duration of oral antibiotics in weeks (mean; SD)	9.2 (5.7)	11.2 (6.4)	7.3 (4.2)	0.049
Preoperative sinus tract (*n*; %)	10 (20.0)	4 (16.7)	6 (23.1)	0.571
Preoperative Joint aspiration (*n*; %)	34 (68.0)	18 (75.0)	16 (61.5)	0.308
Synovial WBC^a^ in G/l (mean; SD)	23.3 (26.9)	26.8 (11.4)	19.9 (32.1)	0.543
Synovial PMN^b^ in % (mean; SD)	78.6 (27.8)	85.1 (19.6)	72.6 (33.4)	0.272
Serum CRP^c^ in mg/dl (mean; SD)	13.1 (10.5)	16.6 (11.47)	9.8 (8.31)	0.020

^a^*WBC* White blood cell count

^b^*PMN* Polymorphonuclear neutrophils

^c^*CRP* C-reactive protein

## Abbreviations

ASA: American Society of Anaesthesiologists
BMI: Body Mass Index
CCS: Charlson Comorbidity Score
CNS: Coagulase-negative staphylococci
DAIR: Debridement, antibiotics and implant retention
DFR: Distal femoral replacement
GN: Gram-negative bacteria
IFR: Intramedullary total femoral replacement
IQR: Interquartile range
JS-BACH: Joint Specific Bone involvement, Anti-microbial options, Coverage of the soft tissues, and Host status
MP: Megaprosthesis
PJI: Periprosthetic joint infection
PFR: Proximal femoral replacement
SD: Standard deviation
TFR: Total femoral replacement
THA: Total hip arthroplasty
TJA: Total joint arthroplasty
TKA: Total knee arthroplasty
